# On Erysipelas

**Published:** 1826-04

**Authors:** P. C. Blackett

**Affiliations:** Surgeon Royal Navy; and Surgeon Extraordinary to His Royal Highness the Duke of Clarence.


### ART. VI.—*On Erysipelas.* By P. C. BLACKETT, M.R.C.S. Surgeon Royal Navy; and Surgeon Extraordinary to His Royal Highness the Duke of Clarence.

MANY years since, I had occasion to direct my attention to erysipelas, a disease then but little known, and yet not well understood. The following notes were made at that time; the cases subjoined are of more recent occurrence. I am induced to publish both, chiefly with a view of directing attention to the treatment of a disease but little subjected to medicine. The practice here inculcated is one which I have always adopted, and generally with success; and, as it differs a little from that usually advised, the cases so managed may not be unacceptable.

Erysipelas is an inflammatory fever of two or three days, (rarely longer,) attended commonly with drowsiness, often with delirium. Pulse generally full and hard. There succeeds redness of the face or some other part, with a continuation of febrile action, tending either to Erysipelas vesiculosum or E. phlyctenodes, to abscess, or to gangrene.

*Species.*—1. Erysipelas vesiculosum. 2. Erysipelas phlyctenodes. 3. Erysipelas infantum.

*Symptoms.*—Rigors, and other indications of pyrexia; great confusion of the head, amounting even thus early to delirium; coma; nausea, vomiting; pulse quick and hard, strong, or small, as the fever may incline to the inflammatory or typhoid kind. The eruption appears about the second or third day; the fever and sickness then abate, though there often remains some degree of both during the progress of the disease. The redness is at first of no great extent, and gradually extends from the spot it first occupied to other adjoining parts, until it has affected the whole head, trunk, or extremities, according to the first point of attack. When the inflammation is confined to the face, the headache continues until the decline of the eruption; the eyelids swell, and the eyes are closed. If the disease be of unusual duration, the eruption passes often from the one cheek to the other, and extends successively over the forehead, scalp, and nape of the neck. As this characteristic redness spreads, it commonly leaves, or at least is much abated in, those parts it had primarily occupied. All parts so affected are subjected also to a degree of swelling, which remains for a longer or shorter time after the disappearance of the redness. When the inflammation has continued for some time, blisters commonly arise, of a larger or smaller size, in patches on the affected part. These contain a thin colourless fluid, which sooner or later escapes. The surface of the skin in the blistered places becomes at times livid and blackish; but this seldom penetrates deeply, or discovers any disposition to gangrene. Where the parts are not blistered, the cuticle, towards the close of the disease, suffers a considerable desquamation. Occasionally the tumor of the eyelids ends in a suppuration of the lids and cornea.

The fever, however, does not always at this period suffer a remission, but at times exacerbates with the spreading and increasing inflammation; is frequently aggravated by increase of coma or delirium, and the patient expires about the ninth or eleventh day. In such cases, it is supposed that the disease is translated from the external to the internal parts. But this affection of the brain is merely a communication from the external disease, made by sympathetic action, as it even continues and increases with the latter.

The eruption suddenly retiring, the patient is affected with nausea, vomiting, anxiety, &c. The erysipelas may again show itself in a different part: then, he finds himself sensibly relieved. If, instead of the eruption re-appearing, the brain or chest should be affected, death ensues within a few hours. These fatal changes occur oftentimes without the least reason for ascribing them either to any error of practice in the physician, or of regimen in his patient. It must, however, be remembered that, when the head is affected, it is not always that the external inflammation recedes: more commonly the violence of the determination is such, that the internal as well as external arteries take at once their share of it, and the brain and external surfaces suffer equally and together. The same consequences ensue on the lungs being attacked.

When the inflammation attacks the extremities, it extends generally towards the trunk; and, when the tumefaction is considerable, the part affected either suppurates or is covered with small vesications, filled with a clear watery humor, similar to those consequent upon a burn: these afterwards dry and desquamate.

At times a malignant species of erysipelas is epidemical; this frequently terminates in gangrene.

There are some constitutions subjected to a very frequently recurring, and, as it were, an habitual erysipelas; and often it is the same part which, at each period, is the subject of the affection. In these patients, the attack is in general very slight. The disease makes its appearance with a roughness, heat, pain, and redness of the skin, which becomes pale on pressure, regaining its colour on such obstruction being removed. There prevails a febrile disposition likewise, and the patient is hot and thirsty. These symptoms continue only for a few days. The surface becomes yellow, the cuticle falls off in scales; and the patient recovers, without further inconvenience.

Sometimes even this habitual and recurrent species of the disease will continue from eight to twelve days, with the greatest violence, and at last terminate by a plentiful perspiration; which may sometimes be predicted from an excessive restlessness, attended with thirst, and anxiety.

The *predisposing causes* are—a cholero-sanguine temperament, a plethoric habit, and previous affections of the same nature.

The *exciting causes* are—cold and excessive heat, or vicissitudes of temperature; abuse of fermented liquors; suppressed evacuations, and all other causes inducing plethora; the presence of irritating matter in the primæ viæ, more especially of acrid bile; wounds; contagion.

The event of this disease may be foreseen from the degree of violence accompanying those symptoms which denote more or less affection of the brain. If neither delirium nor coma take place, it is seldom attended with danger; but these symptoms appearing in an early stage of the disorder, the utmost danger may be apprehended. When, therefore, the fever is merely inflammatory, the eruption of a bright scarlet or red colour, not extending over a large surface, unattended with vesicatións, —the fever, delirium, and coma, diminishing upon the appearance of the eruption, and this soon after assuming a yellow hue, with an abatement of the swelling, &c. we may draw a favourable prognosis of the event. But, if the fever assumes the typhoid form, protracted to the seventh, ninth, or thirteenth day, with an increase of delirium or coma,—the eruption becoming of a dark-red brick colour, suddenly receding from the external to the internal parts, extending continually without leaving its original seat, with livid and dark vesications,—weak, rapid, and irregular pulse,—great loss of strength,—the disease being epidemic,—the patient naturally of a weak habit, or emaciated by previous illness,—the disease being combined with dropsy, jaundice, or other affections, originating in a depraved condition of some organ; then our prognosis will be very unfavourable, and particularly should the disease be consequent upon wounds or operations.

The *treatment* of this disorder must always be regulated by the course its varied nature may pursue; accordingly, our indications will be—

1st. To reduce all inflammatory action.

2dly. To support the strength of the patient, should the disease take on the typhoid form.

3dly. To obviate the tendency to a determination to the head, or any other important organ.

4thly. Neither to meddle with the sloughs, nor puncture the vesications.

When the face is attacked, and there are marked symptoms of phlegmonous inflammation, the antiphlogistic regimen should, in the first instance, be adopted; and, on this being early done, depends its ultimate success. Such depletions as blood-letting and purging will require the utmost nicety and skill in their direction; for it seldom occurs that the fever is merely inflammatory: more generally it is mixed, having the symptoms of synocha in the commencement, and assuming a typhoid character towards the end. Still, in the first stage of the disease, according to the strength and hardness of the pulse and urgency of the symptoms, copious and rapid evacuations of blood will be both necessary and salutary; small and repeated bleedings being, on the other hand, always attended with the greatest danger. Local bleedings are always attended with the greatest peril, as the skin, where it has been punctured, ever disposes to gangrene. Cupping, leeches, &c. should therefore be exploded entirely in the treatment.

When the typhoid form supervenes, the utmost attention must be paid to cleanliness: frequent change of linen, ventilating and fumigating the room of the patient, and frequently sprinkling the apartment with vinegar, is highly necessary; together with the use of fruits, the exhibition of antiseptics, especially cinchona, combined with the mineral acids and sedatives; such as opium, wine, brandy, and bottled porter.

The bowels should be kept continually and freely open during the whole of the disease; still they should not suffer too great an excitation during the last stage. The free exhibition of purgatives during the early period of the attack, is indispensably necessary; and frequently, from the accompanying torpor of the bowels, requires a freedom and liberality of dose in hot climates, that appears alarming in more temperate regions. They should be repeated, and, if occasion requires it, assisted with enemas, till they have produced from ten to twelve copious evacuations. The thorough evacuation of the whole alimentary canal is (I must repeat again) absolutely necessary, and cannot, during the first hours of the attack, be too much insisted on. I have said that, in the latter stage, the bowels should not be too much excited; because a distressing diarrhœa, or constant attempts at evacuation, with tormina, is frequently a most harrassing circumstance at this period. The submuriate of mercury is to be administered in doses of from three to twelve grains, with senna, rhubarb, or any other mild aperient or enema.

Where coma or delirium exists, the semicupium, together with sinapisms and blisters to the nape of the neck, have afforded much relief.

To obviate a tendency to a transfer of this disease to any internal part, must be our next consideration. When this takes place to the stomach, great anxiety and sickness occur; violent and burning pain; sudden and great prostration of strength; small, hard, contracted, and rapid pulse; frequent and distressing hiccup. The pain is much aggravated by taking food, and by pressure; and an erysipelatous eruption appears on the fauces.

When the change is directed to the heart, syncope supervenes, with great anxiety about that region, and a sudden deprivation of animal and vital power and action, the patient being at once deprived of pulse, sense, and motion; the countenance has a death-like paleness, the extremities cold and flaccid; the mouth is sometimes closely shut, sometimes gaping open. Should recovery from this paroxysm take place, it is accompanied with deeply-drawn and heavy sighs.

When the change is to the lungs, there is an acute pain referred to the side, about the sixth or seventh rib, lancinating to the sternum and scapula; the breathing is anxious, and the pain is much increased during inspiration ; frequent, hard, contracted, and vibrating pulse; the face becomes morbidly pale, the extremities shrunk and cold.

When referred to the head, excruciating pain in that part is felt, with extreme sensibility to light and sound ; the expression of the countenance is wild; the face cold, pale, and shrunken, with constant pervigilium. At last, the face becomes flushed and turgid; the eyes stare, and appear as starting from their orbits, with ferocious delirium. The skin is dry and burning; the tongue parched, white, or yellow, or black; pulse small, hard, and rapid.

The treatment here is regulated by the symptoms which lead to a judgment whether this form of the disease be consequent upon typhus or synocha. If it has followed the former, blisters, semicupium, sinapisms, &c. should be advised; if the latter, general and topical bleeding may be permitted ; blisters, fomentations, pediluvium, and the general treatment for inflammation of the organ affected.

The external applications directed are various: starch and flour (which are perhaps the best), litharge, warm spirituous fomentations, and ointment of opium and belladonna. Cold applications should ever be avoided ; even when the patient is young, and the inflammatory action is excessively strong.

When the erysipelatous inflammation produces vesications, suppuration, or gangrene, the practitioner should be careful not to puncture the vesications, or lay open the abscesses, or remove the sloughs, but in every case let nature act for herself: the only thing he has to do is to watch quietly the progress of these terminations. If vesications form, absorbent powders should be applied ; if abscesses, poultices and fomentations; if gangrene, a similar treatment to abscess. But, should these applications be found painful or insufficient, opium or the belladonna may be directed.

The erysipelas of new-born infants must be treated according to symptoms ; but the chief thing to be depended on is a thorough evacuation of those dark-coloured fæces, with which the bowels are constantly loaded.

CASE I.

T. W—, a gentleman about fifty-seven years of age, a very free liver, and of a very gross habit of body, in the autumn of 1821, slipped while stepping into a coach, and bruised the upper part of his right ankle, by which the skin was abraded to about the size of a sixpence. The wound was so slight, that he took not the least notice of it, until four or five days after, when it began to pain him and to swell. He applied to an apothecary, who advised him to apply a preparation of the liq. plumbi acet. and water. This appeared to relieve him until the tenth day, when he was attacked with rigors; the pain of the wounded part became more severe, and considerable swelling took place. Towards bed-time, his head began to ache violently, and the whole body became of a burning heat; the pain of the affected extremity increasing in a most aggravated degree. A poultice was applied, not only to the wound, but up to the very calf of the leg.

The next morning I was called in to attend him. I found him in a state of high delirium; his pulse small, hard, and rapid; his tongue brown; skin dry and burning; bowels constipated; urine scanty, and of a dark red colour; the leg, as well as the foot, was highly inflamed and swollen; vesications had formed. I ordered the poultice to be discontinued; the leg and foot was dusted with starch-powder, and rolled in a calico bandage. The bowels were relieved by the following medicines:

℞. Hydr. submuriat. gr. vj.
Conf. rosæ q. s. ut fiat pil. j. statim sumend.

℞. Infusi sennæ, ℥iss.

Magn. sulph. ʒiij.

Træ. jalapæ, ʒij. M. fiat haustus post horam unam sumendus.

His diet was to consist of weak mutton broth, barley-water, and fruits.

The next day, I found the medicine had operated well; his delirium had abated, and his tongue beginning to clean; pulse less frequent and soft; pain of the foot and leg much lessened; the swelling and redness were also abated, but the vesications were larger, and around their bases the skin had turned to a very dark brown colour, and was excessively irritable to pressure; the skin generally was comfortable and moist. I ordered a continuance of the same applications, with the following:

℞. Infus. cascaril. ℥vj.

Magnes. sulph. ʒij.

Ext. conii, ℈j.

Acid. sulphur. dilut. m. xv. M. fiat mist. cujus capiat coch. iij. tertiâ quâque horâ.

I directed his diet to be full, and that he should drink, during the four and twenty hours, a pint of port-wine.

On the following morning, his general health was much improved; bowels open, tongue clean, no thirst; urine copious and clear; pulse weak, but regular. The vesications were still very brown: one had given way, and discharged a yellowish fluid.

The same diet, external applications, and internal treatment, were persevered in for four days longer. When I again saw him, I found that all the vesications, save one, had subsided: this one was about four inches long, and two broad. He was altogether much improved.—Perstet in usu medicamentorum.

In about three days from this, the vesication gave way; and, in a week, the whole of the skin which covered the vesications came away, leaving the parts very tender for a long time. By continuing the applications, keeping the bowels regular, &c. he became in the course of two weeks perfectly well.

In this case I have not the least doubt that, had bleeding been resorted to, the patient would have died in a few days. The blood would have been found entirely dissolved, and all its particles would have separated themselves in a few minutes subsequent to the removal from the vein. The delirium in this patient was principally produced, no doubt, by the putrid matters collected in the prima viæ; to remove which is a most important object, contributing to mitigate, and often to prevent fatal consequences.

From the very considerable number of cases exactly similar to this, which I have had an opportunity of attending to, I can feel no difficulty in giving my opinion as to the effect of the plan of treatment here pursued. In all these cases, the disease has been attended more generally with an irregular kind of typhus than with synocha; the fever, which always sooner or later took place, partaking decidedly of the characters peculiar to the former. I have found these cases tedious, particularly where the exhibition of tonics was delayed. I have, however, constantly found that, when the tonics—acids, port-wine, and nutritious diet,—have been early and freely used, they have produced the most beneficial effects, and, I may say, certainly cured my patients.

CASE II.

M. P—, a young woman, aged nineteen years, was attacked with erysipelas of the right side of the face, during the summer months of 1822. She was ordered by her medical attendant to apply a cooling lotion, and take a gentle aperient. She got well (it was supposed) about the fourth day; but, on the sixth or seventh from the first attack, the disease appeared, with aggravated vigour, on the left side of the face. The same remedies were had recourse to, but, instead of relieving the symptoms, materially increased them. Two days after this second attack, I was requested to see her, and take her under my charge. I found the face of my patient very much swollen; the eyelids closed and tumefied to the greatest degree, so much so that it was impossible to separate them; and, in the attempt, a sanious discharge began to flow from between them. She complained of great pain and heat in the inflamed parts, which presented the genuine character of erysipelas. She was excessively restless and thirsty, with great heat over her whole body. Her pulse was upwards of 120, and feeble; there was a slight degree of delirium; her bowels were constipated; urine scanty, and of a dark red colour, affording a thick deposit. I immediately gave orders she should have an enema, and prescribed the following:

℞. Hydr. submur. gr. iij.

Extr. conii q. s. ut fiat pil. j. statim sumenda.

℞. Infus. sennæ comp. Aq. menth. pip. āā ℥ijss.

Magnes. sulph. ℥j.

Træ. sennæ comp. ʒss. M. fiat mistura cujus capiat cochlearia duo tertiâ quâque horâ, donec alvus copiose respondeat.

I directed also the application of a bread-and-milk poultice to the inflamed eyelids every three hours.

The next morning, I found that the enema and aperients had freely operated; thirst had abated, and the heat of the body decreased; pulse ninety-six, but still weak; urine not quite so high-coloured, and rather more in quantity; pain much less, but she complained of considerable weakness; had also passed a better night than before. The eyelids were not quite so much inflamed: I endeavoured to open them, but, as yesterday, a sanious discharge, with a few yellow particles of sloughs, followed the attempt.—Ordered the poultice to be continued, and that she should occasionally take a little port-wine negus; her diet to be broths. Prescribed the following draught:

℞. Sodæ carbon. ℈j.

Succi limonis recentis q.s. ad saturationem, efficiendam

Infus. cascaril. ℥iss.

Syr. aurant ʒj. M. fiat haustus quartâ quâque horâ sumendus.

On the following day, I found her much the same, perhaps rather better than otherwise. To continue the medicines and applications as before. Has not had an evacuation: ordered, therefore, the following draught early in the morning:

℞. Pulv. rhæi, gr. x.
Potass. tart. gr. xx.
Aq. menth. virid. ℥iss. M. fiat haustus primo mane sumendus.

To-day (the sixth of the attack) I found her in a weaker state than yesterday; pulse weak and rapid, and tongue furred: the swelling was much subsided, but had acquired a livid colour; bowels had been twice opened; there appears a slight degree of coma; there is also a small slough on the left eye, which, on examination, proves to be a small portion of the cornea. No pain,—but very restless. Gave directions that she should take a pint of wine in the twenty-four hours, with nutritious broths, &c.; the poultice to be continued, and the following taken:

℞. Infus. cascar. ℥vss.
Træ. cinchonæ, ℥j.
Extr. conii, gr. x.
Acid. sulph. dilut. m. xv. M. fiat mistura, cujus capiat cochlearia ij. tertiâ quâque horâ.

This morning (seventh) she could open the eye; but the eyeball was so inflamed that it was impossible to distinguish the pupil, excepting at the place where the cornea had sloughed. Pulse seventy-two; thirst less; excessively weak.—To continue all prescriptions excepting the poultice; the eye and lids to be kept clean by a warm infusion of chamomile flowers. Wine, with generous diet, to be continued.

To-day (eighth) much as yesterday.

Did not see her for two days: found her then (eleventh day) much improved. There had been a discharge of pus from the inflamed eye and lids; the eyeball had nearly recovered its usual appearance, excepting at the point where the cornea had sloughed.—Ordered the chamomile application to be used cold, and the other remedies to be continued as before.

In five days more, I found her perfectly convalescent. There was a slight protrusion where the slough had taken place: the only thing now complained of was debility. I advised her, when sufficiently strong, to consult an oculist.

I must add, that, during this young lady's sickness, the nurse and servant in attendance were both attacked with erysipelas: strong aperients, and a free use of port-wine, stopped the complaint almost immediately.

This erysipelas phlegmonodes, no doubt, was the production of those repellents which were applied to the early appearance of the disease, it being then probably but of the simple species. Applications of cold lotions may cause a suspension of inflammation, only, however, for the renewal of the attack in a more aggravated form, inducing an irritation which, too frequently, is entirely out of our power to overcome.

Erysipelas was very prevalent during the summer months of 1822, spreading rapidly both in town and country, and was very fatal; no period of life being exempted from its attacks. It was contagious in one family that came under my observation: Mr. ——, his lady, the nurse, and two servants, died. In the public institutions, nearly all those attacked with the disease fell victims to its power; especially those who had undergone any operation. In warm countries, this disposition to become affected with erysipelas after operations is very common.

CASE III.

J. W—, aged twenty-two years, ran a fork into his little finger: he paid no attention to the injury, until a painful sensation in his arm prevented him from using it. Soon it became swollen and inflamed; symptoms of erysipelas came on rapidly. I ordered him hot fomentations and aperients. In two days, the swelling and inflammation subsided in the arm; but the finger, as well as the hand, continued as before. I directed him to poultice the finger. In two or three days, pus was formed at a short distance from the place of the puncture. In five more days, the abscess gave way, and discharged considerably. The tendon was sloughing, and the part very painful; so much so, that he could not bear the poultice. I then ordered him the following ointment and mixture:

℞. Extr. belladonnæ, ʒij.

Cerat. sapon. ʒvj. M. fiat unguentum, nocte maneque applicandum.

℞. Extr. conii, ℈j.
Infus. cascaril. ℥vss.
Acid. sulph. dil. m. xv. M. fiat mist. cujus capiat 4tam partem 4tis horis.

On the following morning, the parts were easy, and all, excepting the tendon, healthy in appearance. Complains that he cannot sleep at night.—Directed the continuance of the former prescriptions, with the following pills:

℞. Extr. rhæi, gr. viij.
Opii gr. jss. M. fiat massa in pilulas ij. hora somni, maneque sumendas.

On the following day, I found the tendon loose, and the upper portion detached. He felt himself better, having had a good night. Bowels open.—Perstat in usu medicamentorum.

Did not see him for two days. At the expiration of which time, the tendon was still adhering, but slightly. In three days, the tendon came away, irritation subsided, and he rapidly recovered.

CASE IV.

A young man, about twenty-six years of age, a carpenter by trade, wounded his right foot with a nail. He was of a delicate constitution. The wound did not bleed, but soon began to assume a black colour, and felt benumbed: this, in about half an hour, was succeeded by lancinating pains. The foot soon began to swell, extending in tumefaction as far upwards as the groin. The next morning it became erysipelatous. I saw him on the following day. The limb was red, and much inflamed; the swelling had extended above the groin; the pain had increased, and extended up the body to the sternum, carrying an uneasy sensation to the throat. Being fearful of lock-jaw, I immediately laid open the wound made by the nail through the fascia: a large quantity of matter was discharged.—I ordered him to poultice the part, and gave him the following mixture:

℞. Magnes. sulph. ʒiij.
Mist. camph. ℥vss.
Træ. opii, m. xxiv. M. fiat mistura, cujus capiat cochlearia iij. tertiâ quâque horâ.

On the next day, I found all bad symptoms had vanished: his bowels open, and the erysipelas subsiding. In the course of three weeks he recovered.

11, *Park-street, Grosvenor-square;*
*February,* 1826.

